# Effectiveness of self-care interventions for integrated morbidity management of skin neglected tropical diseases in Anambra State, Nigeria

**DOI:** 10.1186/s12889-021-11729-1

**Published:** 2021-09-25

**Authors:** Chinwe C. Eze, Ngozi Ekeke, Chukwuka Alphonsus, Linda Lehman, Joseph N. Chukwu, Charles C. Nwafor, Eileen Stillwaggon, Anthony O. Meka, Larry Sawers, Joy Ikebudu, Moses C. Anyim, Kingsley N. Ukwaja

**Affiliations:** 1 Medical Department, German Leprosy and TB Relief Association, Enugu, Enugu State Nigeria; 2 American Leprosy Missions, 120 Broadus Ave, Greenville, SC 29601 USA; 3 Department of Economics, Gettysburg College, Gettysburg, PA USA; 4 Department of Economics, American University, Washington, DC 20016-8029 USA; 5 Department of Internal Medicine, Alex Ekwueme Federal University Teaching Hospital, Abakaliki, Ebonyi State Nigeria

**Keywords:** Self-care, Morbidity management, Leprosy, Buruli ulcer, Lymphatic filariasis

## Abstract

**Background:**

Lymphatic filariasis (LF), Buruli ulcer (BU) and leprosy are neglected tropical diseases (NTDs) of the skin co-endemic in some communities in Nigeria. Not enough is known about the effectiveness of integrated morbidity management and disability prevention in people with these conditions. An integrated self-care intervention was carried out for people with these skin NTDs in two endemic communities of Anambra state, Nigeria. The objective of the study was to assess the effectiveness of self-care practices on costs of care, disability status and health-related quality of life.

**Methods:**

This study utilised a quasi-experimental pre-test/post-test design to assess the effectiveness of the self-care interventions for people affected by NTDs to care for these impairments at home. Data were collected using questionnaires administered at the beginning and at the end of the intervention on monthly cost of morbidity care, and on participants’ disability status and their quality of life (QoL). Focus group discussions (FGDs) were held with both the participants and healthcare workers at follow-up.

**Results:**

Forty-eight participants were recruited. Thirty participants (62.5%) continued the self-care interventions until the end of the project. Of those, 25 (83%) demonstrated improvement from their baseline impairment status. The mean household costs of morbidity care per participant decreased by 66% after the intervention, falling from US$157.50 at baseline to US$53.24 after 6 months of self-care (*p* = 0.004). The mean disability score at baseline was 22.3; this decreased to 12.5 after 6 months of self-care (*p* < 0.001). Among the 30 participants who continued the interventions until the end of the project, 26 (86.7%) had severe disability score (i.e. a score of 10–46) at baseline, and the number with severe disability fell to 18 (60%) of the 30 after the intervention. The mean QoL score increased from 45.7 at baseline to 57.5 at the end of the intervention (*p* = 0.004).

**Conclusions:**

The 6-month self-care intervention for participants affected by BU, leprosy, or LF led to lower costs of care (including out-of-pocket costs and lost earnings due to morbidity), improved QoL scores, and reduced disability status.

**Trial registration:**

ISRCTN Registry: ISRCTN20317241; 27/08/2021, Retrospectively registered.

**Supplementary Information:**

The online version contains supplementary material available at 10.1186/s12889-021-11729-1.

## Background

Globally, over one billion people are affected in tropical and sub-tropical countries by a diverse group of diseases and conditions collectively called neglected tropical diseases (NTDs). NTDs are commonly found in settings with a high level of poverty, poor living conditions, and poor sanitation [1, 2]. They are often associated with physical impairment and disability, accounting for 25% of Disability Adjusted Life Years globally [3, 4]. Most of the 20 NTDs are endemic in Africa. They can be grouped into 3 categories depending on whether their effective control can be achieved through mass delivery of preventive chemotherapy (e.g. onchocerciasis, schistosomiasis), intensified disease management (e.g. Chagas disease, leishmaniasis), or both measures, as in the case of lymphatic filariasis (LF), trachoma and yaws [1–4].

Some NTDs have cutaneous manifestations that are associated with physical changes and losses to body structures causing permanent impairments and disability. Examples include Buruli ulcer (BU), leprosy, mycetoma, cutaneous leishmaniasis, yaws, and LF, which can manifest with ulcers, scars, contractures, subcutaneous nodules, lymphoedema, severe itching, and impairments in mobility [5]. The frequent co-endemicity of these NTDs and similarities in their clinical presentation and care call for their integrated management, especially where financial and human resources are scarce [6]. In 2013, the World Health Assembly and World Health Organization Regional Committee for Africa adopted resolutions that recommended an integrated strategy for the management of NTDs [7, 8]. Recently, the World Health Organization (WHO) published a training guide on cutaneous NTDs for front-line health workers to help countries adopt the integrated strategy [9].

Although an integrated approach to control of skin NTDs could potentially reduce transmission, shorten delays in diagnosis, and lower costs for both participants and the health systems [5], little is known about the effectiveness of integrated skin-NTD surveillance and interventions on participant costs, disability status, or health-related quality of life (QoL). Some interventions for morbidity management and disability prevention for people with LF morbidity have shown economic benefits, reduction in disability, and improvement in their QoL [10–13]. Also, integration of NTD surveillance was successful in improving case detection for LF and podoconiosis in Ethiopia [14], and for BU and leprosy in Benin [15]. A study in Nepal assessed the feasibility of integrating LF patients into existing leprosy self-help groups and found that participants’ attitudes towards integration were positive [16]. Nevertheless, we could not find any study that focused on the integration of morbidity management and disability prevention for more than two skin NTDs.

Nigeria has the highest burden of NTDs in Africa and accounts for a substantial proportion of the global burden. Thirteen NTDs are endemic to Nigeria [17]. There is a need to improve morbidity management of NTDs in the country. The WHO has called for research on piloting the integrated morbidity management approach [5]. Evidence to guide the process in Nigeria is crucial but lacking. This paper reports on a pilot project of a community self-care intervention for morbidity management and disability prevention (MMDP) for participants with leprosy, BU, or LF. and assessed the effectiveness of the intervention on participants’ costs, disability status, and health-related quality of life.

## Methods

### Study design

This study utilised a quasi-experimental pre-test/post-test design to assess the effectiveness of the self-care intervention in the two rural communities of Okpoko and Ogbakuba in Ogbaru Local Government Area, Anambra State, Nigeria from December 2017 to June 2018 for participants with impairments from BU, leprosy or LF. Participants were recruited between December 2017 and March 2018. The intervention period of the study lasted for 6 months from December 2017 to May 2018. Final post intervention assessment was conducted in June 2018.

### Study setting

The project was carried out in Okpoko and Ogbakuba, two rural communities in Ogbaru LGA of Anambra State, Nigeria. The Ogbaru LGA has a population of nearly 300,000. It is located within a tropical rainforest belt that is co-endemic for onchocerciasis, lymphatic filariasis, Buruli ulcer, leprosy, schistosomiasis and soil-transmitted helminthiasis [18]. Three of those NTDs (LF, BU, and leprosy) have primary cutaneous manifestations with substantial morbidity and disability and were the target of this study.

Four primary healthcare centres involved in the treatment and control of BU, leprosy, and LF were located in the two communities. Furthermore, participants in a December 2016 focus group discussions in the two communities had expressed an interest in being able to care for themselves and reduce their healthcare costs. Given that interest and expertise were present in the two communities, they were chosen as the main project sites.

### Participants and recruitment

**Fig. 1:**
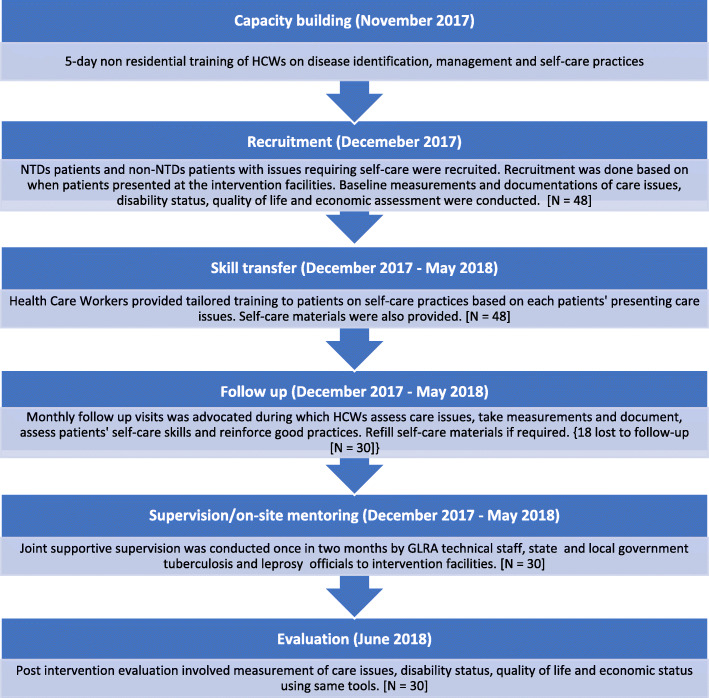
Flow chart describing the approach of the research and study participants

The study included individuals diagnosed with LF, BU or leprosy who had completed specific treatment for their condition but who still had morbidities requiring additional care. In Nigeria, the diagnosis of LF is clinical [18], while leprosy and BU are diagnosed according to national and international guidelines [19, 20]. Briefly, leprosy is diagnosed by finding at least one of the following cardinal signs: loss of sensation in a pale (hypo-pigmented) or reddish skin patch, a thickened or enlarged peripheral nerve with loss of sensation and/or weakness of muscles supplied by that nerve, or the presence of acid-fast bacilli in a slit skin smear [20]. BU is diagnosed by taking a swab/aspirate/tissue from the lesion and conducting laboratory evaluation using one or more of the following: examining a smear after staining by the Ziehl-Neelsen method, culture, or polymerase chain reaction [19, 20]. In the present study, the clinical diagnosis of participants with LF was re-confirmed at presentation. Those previously managed for BU and leprosy whose records were available in the laboratory or in treatment registers of the facility where they were managed were considered to have had laboratory-confirmed BU or leprosy. We recruited all available and consenting participants previously managed for LF, BU and leprosy who presented with specific morbidities such as ulcers, contractures, swellings, stress ulcers within healed scars, and limitations of movement, in one of the study’s primary health centres. During recruitment of participants, we observed a few individuals who were previously not treated for any of these three diseases or whose disease had spontaneously healed presented with one of the morbidities targeted by this project. Given that we were piloting an integrated morbidity management project in the community, these individuals were also recruited into the study despite their lack of formal diagnosis or previous illness. Figure 1 shows a flow chart describing the approach of the research and study participants. As this was a pilot study, we performed no formal sample size estimations.

#### Organization of leprosy, Buruli ulcer and lymphatic filariasis disease management

Management of leprosy and BU in Nigeria is integrated through the National TB, Buruli ulcer and Leprosy Control Programme (NTBLCP) [20]. However, not all of the four health centres offer treatment and care for BU. In endemic communities, the NTBLCP in conjunction with development partners such as the German Leprosy and TB Relief Association (DAHW) established facilities for the diagnosis and treatment of BU. Furthermore, prevention of impairments and disabilities (POID) in people affected by leprosy and BU generally involves some or all of the following: early case detection and adequate disease specific treatment, preservation of nerve function, preservation of vision, preservation of mobility, ulcer management, provision of protective footwear, and self-care training. Although training participants in self-care is part of the POID activities recommended by the NTBLCP guideline [20], this is mainly focused on educating the participants on daily inspection for signs of injury, use of protective footwear, general hygiene and good nutrition. Activities like management of cracks and ulcers, guidance to prevent or reduce limitations of movement through exercise and activity, and individual or group self-management of swelling (oedema) were not emphasised.

Management of lymphatic filariasis (LF) at the national level is under the coordination of the National NTD Programme. Also, while Nigeria has achieved much with community-directed preventive chemotherapy for LF, guidance and interventions to manage lymphoedema and hydrocele in the country are lacking. The general advice is for those affected by lymphoedema and hydrocele to be referred to a nearby health facility for morbidity management, but the capacity to meet the needs of those affected by LF is inadequate [19]. The foregoing show that the management of cutaneous NTDs in the region is spread across numerous agencies and groups. The objective of this study was to experiment with integrating care across NTDs and to take advantage of synergies and scale economies.

#### Self-care project for morbidity management of Skin NTDs

In the study setting, the cadre of healthcare workers involved in the project are community health extension workers and community health officers. The self-care intervention for integrated morbidity management of skin NTDs had the following components:

- Improvement of healthcare workers’ (HCWs) knowledge and skills to identify and manage NTD impairments and complications locally or refer participants to other individuals or organizations that could help. This involved a 5-day non-residential training of HCWs in integrated management of NTD impairments using the American Leprosy Mission’s monograph, “*Ten steps: A guide for health promotion and empowerment of people affected by NTDs”* [21]. The training involved the identification and management of common impairments and teaching self-care to affected participants. In addition, HCWs received guidance on measuring and recording impairments (for example, ulcer size, size of swelling, and limitations of movement) during participants’ baseline and follow-up visits at the health service. Six HCWs (who were community health extension workers and community health officers) from four primary health centres in the study sites were trained in integrated morbidity management and disability prevention for skin NTDs.
- HCWs’ provision of health education about NTDs and teaching participants with NTD self-care skills on how to manage their own impairments at home. This involved HCWs working with each participant to identify and care for his/her impairments so that they could manage their own care. The skills taught to participants during monthly visits with HCWs included skin and scar care, wound dressing, guidance on sterilisation of bandages, management of swelling and prevention and management of movement limitations. Monthly visits were not feasible for participants who lived too far away, but every participant had at least two visits. During these visits, the HCWs evaluated the participants’ impairments, addressed concerns or challenges faced by the participant when implementing self-care at home, and recorded changes in participants’ impairments.
- Improvement in self-care practices among participants with skin NTDs. All participants in the study had BU, leprosy or LF with impairments and movement limitations or had similar impairments not related to NTDs (such as diabetic ulcers, sickle cell disease and trauma). Welcoming participants without NTDs was important to increase uptake and reduce resentment among those who otherwise would have been turned away. All of those who wanted to participate were trained at the health facility on self-care specific to their needs using the “10-steps” monograph. Enrolled patients were expected to report to participating health facilities at least once every month for reassessment of impairment, reinforcement of self-care teaching and refill of self-care materials where necessary. In order to evaluate the success of the project, participants were asked to report monthly costs of their impairment care, disability status and Quality of Life (QoL) at baseline and follow-up after the intervention.

#### Instruments and data collection

A standardised questionnaire was used for data collection. The questionnaire consisted of four parts: socio-demographic characteristics, economic costs, disability status and quality of life. The economic cost component of the questionnaire was developed for the study. It allowed the participants to report both out-of-pocket costs of managing their impairments, earnings lost because of their impairments and the costs and earnings lost incurred by caregivers. Earnings lost was estimated from the difference in self-reported hours of work done by the patient and their estimate of the time a healthy person in the same job or similar job will do the work. Similarly, the time lost by a caregiver in taking care of the patient was documented. In estimating the financial equivalent of the earnings lost by the patients and time lost by the caregivers, we assumed that an average unskilled worker in Nigeria would work 20 days a month, 8 h per day (160 h a month), we used the minimum wage rate in Anambra State to derive an hourly wage rate for estimating lost earnings and time.

Disability status of each participant was assessed at baseline and at follow-up using the World Health Organization Disability Scale-2 (WHODAS-2.0) [22–24]. The WHODAS 2.0 instrument consists of 12 items covering different disability domains including standing and walking, household tasks, learning and concentration, washing and dressing oneself, maintaining friendships, emotional functions, and working ability over the previous 30 days. Each of the 12 items is rated on a 5-point Likert-type scale with a score of 0 to 4, where 0 means no disability, 1 means mild, 2 means moderate, 3 means severe, and 4 means extreme or complete difficulty. The total score of WHODAS 2.0 is the sum of the 12 sub-scores, ranging from 0 to 48, with lower scores indicating lower disability and better functioning. Total scores of 0 indicate no disability, 1–4 indicate mild disability, 5–9 moderate disability, and 10–48 severe disability [23, 25].

The QoL of participants was assessed using the World Health Organization Quality of Life-BREF (WHOQOL-BREF) tool [26]. The WHOQOL-BREF instrument contains 26 items of which two items evaluate overall quality of life and satisfaction with health. Also, there are 24 additional items clustered into four domains (physical health, psychological health, social relationships, and environment domains) [26]. The instrument contains five Likert-type scales of 1 to 5 with higher scores indicating higher QoL. The mean score in each domain indicates the individual’s perception of their satisfaction with each aspect related to QoL. Higher scores indicate better QoL. The mean score of items within each domain is used to calculate the domain score. The domain scores obtained were then transformed to make them comparable to the scores used in the WHOQOL-100 questionnaire [26]. The WHOQOL-BREF and WHODAS-2.0 have been used in other settings to evaluate disability or QoL of participants with skin NTDs [27] and have been found to give accurate and internationally comparable data as measures of NTD-related morbidity and disability [28]. The full questionnaire was administered at baseline during recruitment of participants for the project (Additional file 1) and at the end of the self-care intervention (Additional file 2).

There were male participants in one focus group discussion (FGD), female participants in two FGDs and HCWs in one FGD. FGDs were conducted to gain insights from the participants and HCWs concerning the effectiveness of the self-care intervention on integrated morbidity management. The FGDs focused on constraints to implementation of self-care at home, personal experiences with the impact on impairments, and prospects for improving the intervention locally.

An integrated morbidity management register was developed to reflect at a glance the health service care burden to be addressed. The registry included the participant’s NTD or other condition, their disease and impairment care issues, and whether or not referral was needed. A disability monitoring card was used by HCWs to record participants’ impairment status at baseline and at follow-up. Wound diameters and oedema were measured and number of joints with limitation of movement was noted and recorded. The clinical data collected between baseline and monthly follow-up visits were important to HCWs in guiding the care provided for participants but were not incorporated into the analysis presented below.

### Data analysis

The questionnaires were entered, cleaned and analysed using SPSS version 22 (Armonk, NY: IBM Corp. USA). Continuous variables were summarized as means ± standard deviation (SD) and categorical variables as proportions. Categorical outcome variables before and after self-care were compared using the McNemar’s χ^2^ test for proportions. The mean costs, disability scores, and QoL scores before and after the self-care project were compared using the paired *t*-test; the difference in means of these outcome variables according to the demographic characteristics of the participants were compared using the Student’s *t*-test. A *p*-value < 0.05 was considered statistically significant. The FGDs were transcribed and analysed using coding and a thematic framework for analysis. The initial meaning units were used to construct themes and subthemes and to develop written descriptions of the experiences. Disagreements in interpretation were reviewed and decided by consensus among members of the research team. For each theme, quotations from participants that seemed especially representative or interesting were recorded and reproduced below.

## Results

### Socio-demographic characteristics of the respondents at baseline

Forty-eight participants were recruited at baseline. The socio-demographic characteristics of the respondents are as shown in Table 1. About 71% (34) of them were aged 20 to 60 years. The mean age of all participants was 42.1 years. Thirty (62.5%) participants were female, 24 (50%) were Catholics, and 29 (60.4%) were married. Also, 44 (91.7%) had completed at least 6 years of formal education and 21 (43.8%) lived in a household with a monthly cash income of US$87.0. A total of 25 (52.1%) of the participants had BU, 19 (39.6%) had other ulcers like diabetic ulcers and other chronic ulcers, 3 (6.3%) had LF, and 1 (2.1%) had leprosy (Table 2). Of the 30 participants who completed self-care, 21 were previously laboratory-confirmed NTD cases (1 LF, 1 leprosy and 19 BU), while 9 were non-laboratory confirmed NTD cases or participants who had other conditions with impairments that could profit from self-care. Other aspects of the sociodemographic characteristics of the respondents are as shown in Table S1.

**Table 1:** Socio-demographic characteristics of the baseline study population, Anambra State

Variables	n (Total = 48)	%
Age group (years)		
≤ 20	6	12.5
21–40	20	41.7
41–60	14	29.2
≥ 61	8	16.7
Gender		
Male	18	37.5
Female	30	62.5
Religion		
Catholic	24	50.0
Protestant	17	35.4
Traditional religion	1	2.1
Other	6	12.5
Marital status		
Married	29	60.4
Never married	13	27.1
Separated	1	2.1
Widowed	5	10.4
Education		
No formal education	4	8.3
Primary	15	31.3
Secondary	26	54.2
Tertiary	3	6.3
Occupation		
Employed	24	50.0
Unemployed (health reasons)	17	35.4
Unemployed (other reasons)	1	2.1
Student	6	12.5
Household income monthly		
No cash income	27	56.3
Irregular cash income	18	37.5
Regular cash income	3	6.3
^a^Mean (SD) household cash income (US$)	87.00 (118.90)	

^a^denotes mean household cash income for the 21 subjects who reported having a regular or an irregular income

**Table 2:** Participants’ diagnosis at baseline and at completion of the self-care project, Anambra State

Disease	Baseline (***N*** = 48)	Completed self-care (***N*** = 30)
Buruli ulcer	25 (52.0)	19 (63.4)
Leprosy	1 (2.1)	1 (3.3)
Lymphatic filariasis	3 (6.3)	1 (3.3)
Others (Diabetic foot ulcers, Sickle cell ulcer, trauma)	19 (39.6)	9 (30.0)

### Summary of care issues/impairments of participants at baseline

Most of the participants presented with more than one care issue at baseline. Care issues included wound, oedema, limitation of movement, scars, infection, hydrocele, eye problems, and miscellaneous diseases. The most common care issue was a wound, presented by 79% of participants, followed by oedema (52%) and limitation of movement (42%) (Table 3).

**Table 3:** Summary of care issues reported by participants on presentation, Anambra State (*N* = 48)

Care issue	n (%)
Wound	38 (79)
Oedema	25 (52)
Limitation of movement	20 (42)
Skin and Scar	17 (36)
Infection	8 (17)
Eye and Vision	1 (2)
Hydrocele	2 (4)
Treatment of disease	2 (4)

NB: A majority of the participants presented with more than one care issue

### Summary of physical limitations, economic costs, disability status and quality of life at baseline

Table S2 shows the individuals’ self-reported limitation pattern. A total of 35 (72.9%) participants indicated that they had moderate to severe limitations. In addition, 37 (77.1%) indicated that they had changed jobs as a result of their illness resulting in a mean monthly reduction in income of US$10.80. Furthermore, 23 (47.9%) of the respondents reported lost work time due to their skin problems, resulting in a mean loss of 9.5 workdays in the month preceding the survey. The majority of respondents lost some workdays due to either travel for treatment-seeking (39.6%) or pain (29.2%) from their skin problems. More than half (54.2%) of participants had no regular employment and only 18 of 48 participants usually worked 5 or more hours per day. They also reported that their caregivers lost a mean 4.0 h of time off from work to care for them, and lost a mean 2.1 days off work to care for them in the preceding month.

Table 4 shows the baseline costs for care and earnings lost in the month preceding the survey. Nearly three-quarters of total costs incurred by the participants were out-of-pocket. Half of out-of-pocket costs were for care-seeking visits to traditional healers (51.7%). Other important expenditures were transportation costs incurred during such visits or visits to other health facilities (8.5%), wound-care materials (7.6%) and the cost of purchasing analgesics (11.2%). The mean out-of-pocket cost for morbidity management incurred by the participants was US$86.54 per month. Participants lost earnings due to illness-related job change and lost days due to disability. The combined mean monthly earnings loss was an average of US$38.38 per participant. Thus, the mean participant cost per month was US$124.92. The mean lost earnings of caregivers were US$7.57. For the household including the caregiver, the mean total morbidity management costs per participant was US$132.49.

**Table 4:** Baseline costs among the study participants, Anambra State (*N* = 48)

Type of cost	Participants reporting expenditure N (%)	Mean (SD) costs in US$	Cost (% of total)
**Out-of-Pocket Costs**			
Fetching clean water for chores and self-care	14 (29.2)	2.71 (4.88)	3.1
Persons to assist with your work	8 (16.7)	3.02 (8.82)	3.5
Getting persons to care for your children	0 (0)	0 (0)	0
Transportation to clinic/doctor/traditional healer	32 (66.7)	7.36 (11.24)	8.5
Transportation costs to work/school	5 (10.4)	0.41 (1.94)	0.5
Transportation costs to/from market	7 (14.6)	0.99 (3.32)	1.1
Transportation costs to/from social events	12 (25)	1.37 (2.94)	1.6
Self-care materials (soap, clean clothing, etc)	30 (62.5)	3.19 (7.23)	3.7
Wound care materials	28 (58.3)	6.56 (9.33)	7.6
Traditional healers	18 (37.5)	44.74 (124.0)	51.7
Caregiver/helper	10 (20.8)	2.94 (6.65)	3.4
Painkillers (or analgesics)	34 (70.8)	9.72 (17.02)	11.2
Antibiotics	13 (27.1)	2.34 (5.70)	2.7
Antifungal	3 (6.3)	0.10 (0.45)	0.1
Special footwear / clothing	2 (4.2)	0.22 (1.08)	0.3
Mobility assistance / devices	3 (6.3)	0.87 (3.75)	1.0
**Mean out-of-pocket costs**		86.54 (134.22)	
**Earnings Loss**			
Mean (SD) income loss due to job change per month		10.80 (40.11)	
Mean (SD) income loss due to inability to work in the previous month		27.58 (35.71)	
Total mean earnings loss		38.38 (54.43)	
**Total Patient Costs**		124.92	
**Caregiver Costs**			
Mean (SD) value in US$ of time lost to care for participant (hours 4.02)		1.46	
Mean (SD) value in US$ of workdays lost to care for participant (2.1 days)		6.11	
		7.57 (13.87)	
**Total Household Costs**		**132.49 (151.67)**	

1$US = N310 Naira

Table S3 shows the baseline disability status of the participant. The overall mean (SD) WHODAS score for all the participants was 19.9 (11.1). Higher levels of disability were seen in the emotional function 2.4 (1.1), walking 2.4 (1.4), difficulty in day-to-day work ability 2.0 (1.4) and standing 2.0 (1.6) domains (Table S3). Overall, there were no statistically significant gender differences in the total disability (WHODAS) score or in the scores in any of the domains (*P* > 0.05; Table S3). Altogether, 37 (77.1%) of the participants affected had a severe disability score, 7 (14.6%) had moderate disability and 3 (6.2%) had mild disability score at baseline (Table S3). The baseline quality of life (QoL) scores using the WHOQOL-BREF (transformed to 100 scale) are as shown in Table S3. Higher scores indicate higher QoL. The mean (SD) overall QoL score was 49.0 (15.0). There was no gender difference in the mean (SD) QoL score (48.9 ± 13.9 for men vs. 49.1 ± 15.9 for women; *p* = 0.96). The most affected QoL domains among the participants were the physical domain (with a mean (SD) score of 45.6 (20.9)) and the psychological domain (with a mean (SD) score of 45.9 (19.1)). The least affected QoL domain was the social domain with a mean (SD) score of 54.2 (21.6).

### Differences in the costs, disability and quality of life scores of participants who were lost to follow-up

Of the 48 people who were recruited at the beginning of the project and taught self-care skills for their skin NTDs and impairments, 18 (37.5%) ceased attending the self-care health facility mentoring visits and were lost to follow-up. The 30 who remained were mentored during the project and surveyed again at the end of the intervention. Table S4 summarises the baseline mean (SD) household costs, income, disability and QoL scores of participants who maintained self-care practices and those who were lost to follow-up. The mean (SD) monthly household costs of NTD care at baseline for the participants who continued with self-care was US$157.50 (180.33) compared with US$90.79 (71.94) among those who were lost to follow-up, but the difference in the means was not statistically significant. The mean (SD) disability score of participants who maintained self-care practice was 22.3 (9.7) compared with 15.9 (12.3) among those who were lost to follow-up. This difference was not statistically significant (*p* = 0.051). Furthermore, the mean QoL score of participants who maintained self-care practice was statistically significantly lower than those who discontinued self-care (*p* = 0.049). In sum, those who reported higher QoL and perhaps those with less bothersome disabilities were more likely to drop out of the self-care programme.

### Changes in individual economic costs and morbidity burden after the self-care project among 30 participants

At the end of the project, 25 (83%) out of 30 participants who continued in self-care practice demonstrated improvement from their baseline impairment status. At baseline 26 (86.7%) participants reported that they were limited by their NTDs and this decreased to 21 (70%) (*p* = 0.180 based on McNemar’s Chi-Square test) at the end of the self-care project (Table S5). The number of participants reporting no or mild limitations increased from 5 (16.7%) to 14 (46.7%) at follow up. The mean income loss per month due to change of jobs resulting from the skin impairment and the decrease in lost workdays per month were statistically insignificant. The number of hours that caregivers spent caring for participants in the previous month fell from 6.1 to 0.5 (*p* = 0.005 based on paired t-test). By the end of the project, participants were employed more hours per day and caregivers were losing fewer working days to care for their charges.

The costs incurred by participants in the study at baseline and at the end of the self-care project are shown in Table S6. The mean out-of-pocket monthly costs of care per participant decreased from US$110.30 at baseline to US$27.28 at follow-up (*p* = 0.01). Also, the mean monthly earnings loss per participant decreased from US$36.08 at baseline to US$25.41 after 6 months of self-care (*p* = 0.395). The overall mean monthly participant costs at baseline was US$146.38, which decreased to US$52.69 6 months after the intervention (*p* = 0.008). Out-of-pocket costs and earnings loss accounted for 70 and 22.9% of all household monthly costs of care at baseline, respectively. Following the intervention, out-of-pocket costs and earnings loss accounted for 51.2 and 47.7% of all household monthly costs, respectively. Overall, the mean household costs of care per participant decreased by 66% following the intervention, falling from US$157.50 at baseline to US$53.24 after 6 months of self-care (*p* = 0.004).

The differences in mean disability scores at baseline and at follow up are as shown in Table 5. The mean disability (WHODAS) score at baseline was 22.3, and that decreased to 9.6 following the self-care project (*p* < 0.001). (Recall that a WHODAS score of 10 or more is considered severe). There was a statistically significant reduction (*p* < 0.05) in disability scores in every domain of the WHODAS scale 6 months after the participants began practicing self-care (Table 5). At baseline, 26 (86.7%) participants had severe WHODAS disability grade and none had a disability grade of zero (0). After 6 months of practicing self-care, the number with severe disability fell to 18 (60%); 2 participants (6.7%) had no disability (Table 6).

**Table 5:** Differences in mean disability scores and QoL scores (100 scale) between baseline and after self-care, Anambra State (*N* = 30)

	Baseline		Self-care		t-statistic	***p***-value
	Mean	SD	Mean	SD		
**Disability item**						
Total score (0–48)	22.3	9.7	9.6	10.1	4.7	< 0.001
Standing (0–4)	2.4	1.5	1.2	1.2	4.3	< 0.001
Household tasks	1.8	1.3	0.6	1.0	3.8	0.001
Learning	2.0	1.3	1.0	1.3	3.8	0.001
Community life	2.2	1.5	1.0	1.4	3.2	0.003
Emotional functions	2.4	1.1	1.2	1.3	4.1	< 0.001
Concentrating	1.9	1.2	0.7	0.8	4.4	< 0.001
Walking	2.7	1.4	1.3	1.6	4.4	< 0.001
Washing oneself	1.1	1.2	0.1	0.3	4.3	< 0.001
Dressing oneself	0.9	1.3	0.0	0.0	4.0	< 0.001
Dealing with strangers	1.2	1.2	0.6	0.9	2.2	0.039
Maintaining friendships	1.3	1.2	0.7	0.8	2.4	0.026
Working ability	2.4	1.3	1.2	1.3	3.6	0.001
**QoL Domain (Scale = 100)**						
Total score	45.7	15.8	57.5	14.9	−3.2	0.004
Physical domain	40.0	18.9	58.5	20.9	−3.7	0.001
Psychological domain	46.3	21.2	60.5	17.0	−2.9	0.008
Social domain	49.4	22.5	54.2	20.2	−1.1	0.283
Environment domain	47.3	12.7	56.8	12.6	−3.3	0.003

Quality of life (QoL) scores transformed WHOBREF-scale

**Table 6:** Overall disability grade at baseline and after self-care, Anambra State (*N* = 30)

	Baseline	Self-care
	n (%)	n (%)
**Disability grade**		
None	0 (0)	2 (6.7)
Mild	1 (3.3)	5 (16.7)
Moderate	3 (10.0)	5 (16.7)
Severe	26 (86.7)	18 (60)

Table 5 also shows the mean QoL scores at baseline and at follow up 6 months later. The mean baseline QoL score was 45.7, increasing to 57.5 at follow-up (*p* = 0.004). There was a marked increase in the physical domain score following the intervention, increasing from 40.0 to 58.5 (*p* = 0.001), in the psychological domain score from 46.3 at baseline to 60.5, and in the environment domain score from 47.3 at baseline to 56.8 (*p* = 0.003). In contrast, the increase in the social domain score following the intervention was not statistically significant (49.4 vs. 54.2; *p* = 0.283).

### Factors associated with economic costs, disability status and quality of life of the participants

Table S7 examines the demographic factors associated with household costs of care among the participants surveyed. At baseline, mean monthly household costs of NTD care did not differ according to age, gender, and educational status, participant’s occupation, marital status and type of NTD (*p* > 0.05). Participants from households with a regular or irregular income incurred higher costs compared to those from households with no defined monthly income (*p* = 0.001). This finding is not surprising since those without income would be less likely to have the money to pay for expenses associated with their skin problems. Participants who were Catholics or Protestants had lower costs than others (*p* = 0.011). On the whole, the costs decreased after self-care, in some cases by more than half, although the decrease wasn’t statistically significant. Overall, after 6 months of self-care (Table S7), there were no statistically significant differences in household costs incurred for morbidity care among different demographic groups or diagnosis (*p* > 0.05).

The within-group changes in disability status from baseline to follow-up according to the demographic characteristics are as shown in Table S8. The disability status at baseline did not vary significantly according to demographic characteristics (all *p* > 0.05). However, after the intervention, younger individuals (≤40 years old) had a significantly lower disability score compared to older participants (*p* = 0.004). Similarly, after the intervention, participants who were never married or were currently married had significantly lower disability scores compared with those who were separated or widowed (*p* = 0.036). However, there were no significant changes in the disability scores after 6 months of self-care according to the participants’ gender, educational status, religion, occupation, and income (*p* > 0.05).

The within-group changes in the total QoL score from baseline to follow-up according to the participant’s demographic characteristics are shown in Table S9. The total QoL of participants at baseline did not vary according to their demographic characteristics (all *p* > 0.05). However, after the intervention, younger individuals (≤40 years old) had a significant improvement in their quality of life score compared to older participants (*p* = 0.02). Similarly, participants who were married or never married had a significant improvement in their QoL scores compared with those who were separated or widowed (*p* = 0.015). Overall, there were no significant changes in the QoL scores after 6 months of self-care according to the participants’ gender, educational status, religion, occupation, and household income (*p* > 0.05). As expected, there was a strong negative correlation at baseline between QoL scores and disability scores (*r* = − 0.65; p = 0.01). Similarly, after 6 months of self-care, there was a stronger negative correlation between QoL scores and disability scores (*r* = − 0.74; *p* < 0.001).

Table S10 summarises the mean economic burden, disability status and quality of life of NTD and non-NTD patients at baseline and 6 months after self-care. Overall, there were no significant differences in the mean household income ($38.50 vs $9.90; *p* = 0.422) at baseline between the two groups. Also, there were no significant differences in the household costs between NTD and non-NTD cases at baseline (*p* = 0.469) and at follow up (*p* = 0.501). Furthermore, there were no significant differences in the disability score between NTD and non-NTD cases at baseline (*p* = 0.277). However, after 6 months of self-care, participants who were NTD cases had significant reduction in their disability score compared to the non-NTD cases (10.1 vs. 18.0; *p* = 0.026). Similarly, there were no significant differences in the overall QoL score between NTD and non-NTD cases at baseline (*p* = 0.328). However, after 6 months of self-care, participants who were NTD cases had significant improvement in their overall QoL score compared to the non-NTD cases (62.8 vs. 45.2; *p* = 0.002).

When these data were further analysed according to specific diseases (Tables S7, S8 and S9), among participants with BU, there were significant differences in the mean household costs ($122.6 vs $59.0; *p* = 0.011), disability scores (21.7 vs 11.2; *p* = 0.002) and QoL scores (49.1 vs 61.8; *p* = 0.021) at baseline and after 6 months of self-care respectively. However, among other causes of ulcers (non-NTD cases) there were no significant differences in the mean household costs ($185.8 vs $50.4; *p* = 0.10), disability scores (23.8 vs 16.1; *p* = 0.09) and QoL scores (39.5 vs 47.1; *p* = 0.17) at baseline and after 6 months of self-care respectively. The changes in household costs, QoL scores and disability scores among patients with LF and leprosy could not be compared due to the small sample size of the participants.

Findings from focus group discussions (FGDs):

Below we describe what we learned about the interventions, grouped into four major themes.

#### Theme 1. Loss to follow-up

One of the key themes that emerged revealed the reasons for loss to follow-up. Some HCWs believed that some participants abandoned the self-care intervention due to its perceived lack of efficacy.

“Some LF patients were misinformed that self-care practice do not offer cure, making them to abandon the self-care project” (FGD of Healthcare worker).

#### Theme 2. Disability, quality of life and costs

Participants reported a remarkable reduction in their impairments and improvement in their QoL. Almost all the participants who participated in the FGDs found clear linkage between their self-care and improvement in their disability status, QoL and reduction in monthly health costs for care. Here are some examples of participants’ comments:

“Since I was taught how to clean, bandage and dress my wound, I testify that it (the lesion on my leg) is healing. I prefer dressing it myself due to financial constraints” (FGD of participants).

“Please, I believe there is a need to increase community sensitisation about the self-care programme to get more people (who need it) involved. Before I started the self-care programme, I could not walk or stand. In fact, I was carried into the vehicle while going to the hospital, but now I can walk even without a walking stick – all to the glory of this programme. I like it, I benefited from it and I will like it to be extended” (FGD of participants).

“I started treatment somewhere else, but the lesion (ulcer) could not heal. The traditional healers I visited could not do much. Then this programme came, taught me how to treat myself, do the exercises...and now I am completely healed” (FGD of participants).

“Before I started the programme, my lesion (ulcer) had bad odour and I am socially not welcomed, but now it is healing, there is no more odour, and now I can associate freely” (FGD of BU and LF participants).

“I tested positive for BU, I started dressing the wound myself after being taught how to do it. Now, I am completely healed” (FGD of BU and LF participants).

“I was taught how to do exercise that will help me. Before I started doing the exercise, I experienced pains, but with exercise, I no more feel pains” (FGD of BU and LF participants).

“I stopped going to school because of the wound and odour...So I was asked to stop. Now I have returned to school and still have my friends back” (FGD of participants).

“Before now, I could not use my fingers because they were stiff. Now, I can wash plates and sweep, although I am yet to start washing clothes. The other place I went for treatment did not benefit me” (FGD of participants).

“Why I am so happy is because someone like me that used to be very active got confined to the bed due to this ailment. But with this project, I have regained my ability to do some of the things I could not do before” (FGD of participants).

“Before, I do not walk around or stand while bathing. A chair is kept for me at the bathroom to sit while someone assists me with bathing. However, with the exercises I was taught, I can walk from here to the junction, stand while bathing and bath myself too. There is a great difference now” (FGD of participants).

“One of my children stopped school because he had to assist me at home due to my condition, but with the improvement so far, my child has gone back to school” (FGD of participants).

#### Theme 3. Improving on the self-care project

Some participants suggested ways to make the self-care experience more effective. A number of participants recommended frequent group meetings in order to share experiences and challenges in the practice of the self-care at home. Some participants suggested that they needed analgesics and multi-vitamins to assist them in the healing process.

“In the sterilisation of the gauze/bandages for re-use, the sterilisation process of the gauze using heat was challenging especially the bad odour that comes with the process,............. another patient shared his experiences on how she addressed such individual challenges of using heat to sterilise the materials” (FGD of participants).

“I feel we need to organise this type of meetings regularly so that we could draw strengths from the experiences of each other and it will help us better understand the self-care process” (FGD of participants).

“Although I try to practice all the skills I was taught by the self-care project, the pain that comes with it is severe. Please can you also recommend some painkillers for me?” (FGD of participants).

“We need drugs that will prevent that wound from smelling, I receive insults due to the bad smell of my lesion (wound)” (FGD of participants).

#### Theme 4. Prospects

Many participants and HCWs suggested that a booklet or a self-care guide would help sustain the self-care practices. The majority agreed that a booklet or table-top flip chart containing pictures or drawings of self-care exercises for each type of impairment was needed to reinforce what was learned in meetings.

“I really feel that a booklet or guide will be useful in sustaining the self-care practices in our community. With such a book, even someone who is an illiterate can easily get someone or their children to read the booklet for them. It will serve as a reminder /guide” (FGD of participants).

“I also agree that there is a need for a self-care guide. Given that this document is very important, some of us may put the document in our boxes without looking at it again. I feel that rather than a booklet, a table-top flip chart will be more effective as this will allow us to quickly flip to our specific impairments/lesions and review/adopt methods to sustain the self-care needs of our lesion” (FGD of healthcare workers).

## Discussion

It has been recommended that activities for the control of NTDs could be integrated at four levels: disease mapping and surveillance, clinical diagnosis and treatment, community control of mass drug administration, and morbidity management and disability prevention [5, 6]. Reports of these integration efforts have focused on disease mapping and surveillance [14, 15, 29], clinical management [30, 31], or community-directed mass drug administration [32, 33]. To our knowledge, our study is one of the first attempts to implement integrated morbidity management and disability prevention for multiple skin-NTDs using a community-based programme to promote self-care. Here, we report our experience with a pilot project of integrated morbidity management of people already affected by BU, leprosy and LF in a rural district of southeast Nigeria in accordance with the resolutions of the 63rd World Health Assembly and the WHO Regional Committee for Africa [7, 8]. We used the 10-steps monograph and the approach proposed by Mitja et al. [5, 21]. The study showed that the integrated six-month pilot self-care intervention for NTDs with cutaneous manifestations and some non-NTDs with similar impairments substantially lowered personal costs for care of impairments, reduced perceived disability and improved the participants’ QoL.

In this study, 2, 6 and 92% of the participants had leprosy, LF, or BU, respectively. The very low proportion of leprosy and LF cases at the baseline was because there was no active case search for individuals affected with these diseases in the communities or maybe incidence in the communities was low.

Thirty-eight percent of baseline participants were lost to follow-up. Those who dropped out of the programme had significantly higher baseline QoL scores (*p*-value = 0.049) and lower disability scores that nearly reached statistical significance (*p*-value = 0.051) compared to those who remained in the programme until completion. Those who dropped out also had higher mean family income and lower monthly costs for caring for their disabilities, but those differences in means were not statistically significant. Thus, it appears that the participants who had a greater need for help in managing impairments were more likely to continue in the programme until end-term follow-up. Some health workers reported that a few of the participants were unable to have a follow-up visit due to lack of funds for transportation to the health facility.

At baseline, the majority of the out-of-pocket costs incurred were for care from traditional healers, US$44.70 (51.7%); analgesics, US$9.70 (11.2%); transportation costs used for health-seeking, US$7.40 (8.5%); and for buying wound-care materials US$6.50 (7.6%). This suggests that an intervention that targets patients’ education/training in science-based self-care skills could substantially reduce the patients’ expenditure for traditional healers.

This study found that participants who continued in the self-care programme up till the end of follow up were in households whose mean monthly spending for skin NTDs at baseline was US$157.50. At follow-up, the mean monthly household costs for care had decreased by 66%. Previous studies have shown that those with NTDs incur substantial out-of-pocket costs and lost earnings due to their morbidity [25, 34–36]. The findings of this pilot programme indicate that self-care intervention can substantially lower the economic burden of morbidity care for both NTDs and non-NTDs for participants and their families. This finding is consistent with an earlier report by Stillwaggon et al.*,* who calculated the economic cost with and without community-based LF morbidity management and found that community-based LF management led to a reduction in economic costs to participants with lymphoedema that was more than 130 times the cost of the intervention [13].

This study found that the disability status self-reported at baseline had substantially declined by follow-up across all domains of the WHODAS scale. In particular, 6.7% of the participants who previously had some disability reported none following the self-care intervention. The proportion of participants with severe disability decreased from 86.7% at the beginning to 60% at the end. This suggests that the self-care intervention substantially reduced the participant’s reported disability. There were no differences in disability status according to demographic characteristics or diagnosis. However, following the self-care intervention, it appears that the reduction in disability was greatest among younger individuals and among participants who were married or never married. This difference in disability status according to the demographic profile of the participant following the self-care intervention needs to be explored in further studies. Two studies have shown that WHODAS 2.0 scale has a high level of acceptability, acceptable feasibility, internal consistency, discriminant validity, and construct validity in general [28], and in participants with LF specifically [37]. A community-based lymphoedema management programme in India over a 24-month period demonstrated marked reduction in disability (assessed using WHODAS) and prevented days of work lost [38]. Given the strong negative correlation between the WHODAS 2.0 scores and the WHOQOL-BREF scores, there is a need to include these instruments in the assessment of the impact of morbidity management and disability prevention interventions for skin NTDs in future studies.

This study found a substantial increase in the QoL scores of the participants following the self-care intervention. In particular, the domain-specific increase in QoL scores was significantly higher in the physical and psychological domains of the WHOQOL-BREF with minimal change in the social domain that was not statistically significant. This may be because the project focused on training participants to perform the self-care skills at home individually without a concurrent community sensitisation programme to alter community perceptions about these NTDs and MMDP. Several studies of the role of self-care and home-based care for morbidity management of NTDs with cutaneous manifestations have been conducted among those with LF. A systematic review of these studies demonstrated that hygiene-centred self-care interventions reduced the frequency and duration of acute episodes by 54% in those with LF lymphoedema [39]. Two studies in India and Sri Lanka assessed the impact of self-care intervention for LF lymphoedema after 6 months and 1 year using a LF-specific quality of life questionnaire-LFSQQ and the Dermatology Life Quality Index, respectively. Both studies demonstrated remarkable improvement in QoL following MMDP programmes [10, 11]. Our findings showed that integrated self-care MMDP for participants with BU and LF showed marked improvement in the QoL scores for individuals with these NTDs. There is a need to maintain the use of the generic disability and QoL assessment tools like WHODAS and WHOQOL-BREF in the assessment of the effectiveness of integrated MMDP interventions for NTDs as it will allow comparative analysis across the different NTDs and non-NTDs [28]. Furthermore, our analysis also suggests that improvements in QoL and disability among participants who had had a laboratory-confirmed NTD was higher compared to those who were non-NTDs. This probably indicates that the non-NTDs participants may require additional measures (such as specific treatment of the primary medical condition) to reduce their disability status and improve their QoL scores. The qualitative study findings reinforced our findings of the effectiveness of the intervention for participants requiring management of impairments and identified challenges that needed to be addressed to improve the sustainability of self-care practices within MMDP for skin-NTDs and related impairments.

Our study has a number of strengths and limitations. Our findings strongly support the hypothesis that an integrated self-care intervention for NTDs with cutaneous manifestations as well as non-NTD-related impairments has the potential to reduce substantially morbidity costs, lower disability status and improve quality of life of the participants affected. These findings need to be interpreted with caution as there was no control group. The small sample size of the study is also a major limitation. As this was a pilot/preliminary study, we did not actively search for individuals with BU, leprosy or LF in the study communities. We believe that the direction of effects observed in this study will inform the design of pragmatic trials in settings endemic with these NTDs. The pre-test/post-test design may be prone to residual confounding and temporal changes in the health services during the intervention. However, the follow-up period (6 months) was short and there were no changes in the health services during the project. Other threats to causal validity may be maturation, history, test effects and regression to the mean effects that cannot be controlled when using a single group pre- and post-test design; their overall effect in this study could not be ruled out [40]. However, the triangulation of methods in the study demonstrated improvement and consistent efficacy of the self-care intervention for MMDP.

## Conclusions

In conclusion, we provide evidence that integrated morbidity management through a community-based self-care programme for participants with BU, leprosy and LF lowered health care costs, improved their QoL and reduced disability scores in Ogbaru LGA of Nigeria. We believe that our findings call for additional rigorous, large studies to demonstrate further the impact of self-care interventions within integrated MMDP among persons with skin-NTDs and non-NTDs with similar impairments in Nigeria and other endemic countries. Using the lessons learned, we hope to expand the project of community-based self-care practices that integrate morbidity management for NTDs and non-NTDs with similar impairments combined with a community sensitisation programme that utilizes testimonials from community members who have successfully reduced their impairments through self-care practices. Locally developed flip charts are currently in development and are expected to be a helpful tool to teach the community and remind people of appropriate self-care practices to address common impairments at home and in the community.

## Supplementary Information

**Additional file 1.** Baseline Questionnaire: Assessment of Economic Burden, Disability and Quality of Life of Patients with NTDs in Rural Nigeria.

**Additional file 2.** Endline Questionnaire: Assessment of Economic Burden, Disability and Quality of Life of Patients with NTDs in Rural Nigeria.

**Additional file 3: Table S1**. Access to water supply and environmental sanitation at baseline.

**Additional file 4: Table S2**. Baseline economic cost and burden to participants/family with NTDs and related impairments.

**Additional file 5: Table S3**. Baseline disability quality-of-life (transformed to WHOQOL-BREF 100 Scale) status of the study participants according to gender (*N* = 48).

**Additional file 6: Table S4**. Differences in baseline economic burden, disability status and quality of life of participants who maintained and did not maintain self-care.

**Additional file 7: Table S5**. Morbidity burden of the participants before and after self-care programme, Anambra State (*N* = 30).

**Additional file 8: Table S6**. Costs incurred by participants at baseline and after self-care, Anambra State (*N* = 30).

**Additional file 9: Table S7**. Association between household costs and demographic characteristics of the participants (*N* = 30).

**Additional file 10: Table S8**. Association between total disability status and demographic profile of the participants (*N* = 30).

**Additional file 11: Table S9**. Association between total HRQoL and demographic profile of the participants (*N* = 30).

**Additional file 12: Table S10**. Changes in economic burden, disability status and quality of life of NTD and non-NTD patients who completed self-care.

## Data Availability

The datasets used and/or analysed during the current study are available from the corresponding author on reasonable request.

## Abbreviations

BU: Buruli ulcer
FGD: Focus group discussion
HCWs: Health care workers
LF: Lymphatic filariasis
LGA: Local Government Area
LGTBLS: Local Government Tuberculosis, BU and Leprosy Supervisor
MMDP: Morbidity Management and Disability Prevention
NTBLCP: National TB, Buruli ulcer and Leprosy Control Programme
NTDs: Neglected Tropical Diseases
POID: Prevention of Impairments and Disabilities
QoL: Quality of Life
WHO: World Health Organization
WHODAS: World Health Organization Disability Scale
WHOQOL-BREF: World Health Organization Quality of Life-BREF
