# Supplementary material for: Effectiveness of self-care interventions for integrated morbidity management of skin neglected tropical diseases in Anambra State, Nigeria
Source: BMC Public Health. 2021 Sep 25;21:1748. doi: 10.1186/s12889-021-11729-1 (PMC8465703; doi:10.1186/s12889-021-11729-1)
Supplement: Supplementary file 1 — Additional file 1. Baseline Questionnaire: Assessment of Economic Burden, Disability and Quality of Life of Patients with NTDs in Rural Nigeria. [file 12889_2021_11729_MOESM1_ESM.docx]

**Additional File 1: Baseline Questionnaire**

**Assessment of Economic Burden, Disability and Quality of Life of Patients with NTDs in Rural Nigeria**

**BASELINE (FIRST) SURVEY**

**Code of the Patient _____________ Hospital Number:**………….. .. Date: ___ / ___ / ___

Hello. My name is ________________ and I am working for The German Leprosy/TB Relief Association. We are conducting a survey to improve health services for persons with Skin conditions in your community. This survey will help us to better understand the difficulties people may have due to their health conditions. The information that you provide in this interview is confidential and will be used only for research. The interview will take 15–20 minutes to complete.

I will start with some background questions. **DIAGNOSIS of the patient…………………………**

| **PART ONE: General and Socio-demographic Characteristics of the Respondents** |
| --- |

Please circle the selected options as appropriate

1. **Gender: A.** Male **B.** Female
2. **Age (year)** …………………………….
3. **What is your religion?**
4. Catholic B. Muslim C. Protestant D. Traditional religion E. Other _________
5. **What is your current marital status?**
6. Married B. Never married. C. Divorced D. Separated E. Widowed F. Cohabiting
7. **What is your current educational status?**
8. Illiterate D. Tertiary education
9. Primary education E. Other ____________________
10. Secondary education
11. **What is your current occupation?**
12. Employed C. Housewife E. Unskilled worker G. Student I. Other (specify)…….
13. Unemployed (health reasons) D. Farmer F. Daily wages H. Unemployed (other reasons)
14. **What is your average HOUSEHOLD income per month?**
15. No defined income B. Irregular income of N……. C. Regular income of N…….
16. **What is your source of water for drinking and domestic use?**
17. Tap / Borehole B. Well. C. River / stream D. Other (specify)…………..
18. **What type of sewage disposal system do you have at home?**
19. Pit-latrine B. Water closet C. Cesspit D. Other (specify)…………..
20. **What is the DIAGNOSIS of the patient** (Please confirm from the clinical register, and record below): **………………………………………………………............................**

| **Part TWO: Economic Cost and Burden to Person/Family with NTD** |
| --- |

Now I will like to ask you some questions regarding the costs and how much work/school time you have lost as a result of your skin condition.

| **1. Are you limited in going to school or in the kind of work you can do because of this condition?** | | | | Yes or No | | |
| --- | --- | --- | --- | --- | --- | --- |
| - How are you limited? _________________________________________________ | | | |  | | |
| - Did you have to change work and or choose a different kind of work due to your condition?(circle) | Yes or No | What is the difference in earnings each month? | | | | _________ |
| **2. How much work/school time (days or %) did you lose in the past month due to this condition?** Note: not sure if it is easy to calculate % and if used maybe < 25%, 50%, 75%, 100% | | | | ______Days  ________ % | | |
| - Why did you lose this time from work/school? (treatment, pain, reaction, Acute dermato-lymphangio adenitis (ADLA), hydrocele, wound, surgery, social, other)? _____________________________________________________________________________________ | | | | | | |
| 1. **How many hours of work do you do each day?** | | | | | ____Hrs | |
| - How many hours do others in the same job or similar job work do each day? | | | | | ____Hrs | |
| 1. **How much time per day does your family/caregiver spend taking care of you?** | | | | | ____Hrs | |
| - How many **days** of school did children in family miss in the **past month** to take care of you? | | | | | ____Days | |
| - How much time does your family/caregiver take off work to care for you for this condition each day or month? | | | | | ____Hrs/Days | |
| - What kind of work/employment opportunities does the family/caregiver miss because of providing care? None or missed paid or unpaid work opportunity, what? (household gardening, handcrafts, cooking, caring for children, other_______________________________________________________________________________________ | | | | | | |
| 1. **How much did you spend in the past month on your care and/or additional help due to your condition?** | | | | | | |
| - Others fetching clean water for household chores and self-care activities | | |  | | | |
| - Others doing your work | | |  | | | |
| - Others caring for your children | | |  | | | |
| - Transportation cost to clinic/ doctor / traditional healer | | |  | | | |
| - Transportation cost to work/school | | |  | | | |
| - Transportation cost to/from market | | |  | | | |
| - Transportation cost to/from social events (church, wedding, funerals, other) | | |  | | | |
| - Self-care materials (soap, clean cloths) | | |  | | | |
| - Wound care materials | | |  | | | |
| - Traditional healer/Doctor | | |  | | | |
| - Caregiver help | | |  | | | |
| - Pain killers | | |  | | | |
| - Antibiotics | | |  | | | |
| - Antifungal | | |  | | | |
| - Special footwear or clothing | | |  | | | |
| - Mobility assistance and/or Mobility devices (crutches, canes, wheelchair) | | |  | | | |
| - Other, what?__________________________________________ | | |  | | | |

| **Part THREE: Assessment of Disability Status of NTD patients** |
| --- |

Now I will like to ask you some questions regarding how your skin problem affects your activity of daily living. *(Please circle number of the selected option for each question)*

| **In the past 30 days, how much difficulty did you**  **have in:** | | None | Mild | Moderate | Severe | Extreme or cannot do |
| --- | --- | --- | --- | --- | --- | --- |
| 1 | Standing for long periods such as 30 minutes? | 1 | 2 | 3 | 4 | 5 |
| 2 | Taking care of your household responsibilities? | 1 | 2 | 3 | 4 | 5 |
| 3 | Learning a new task, for example, learning how to get to a new place? | 1 | 2 | 3 | 4 | 5 |
| 4 | How much of a problem did you have joining in community activities (for example, festivities, religious or other activities) in the same way as anyone else can? | 1 | 2 | 3 | 4 | 5 |
| 5 | How much have you been emotionally affected by your health problems? | 1 | 2 | 3 | 4 | 5 |
| 6 | Concentrating on doing something for ten minutes? | 1 | 2 | 3 | 4 | 5 |
| 7 | Walking a long distance such as a kilometre [or estimate equivalent]? | 1 | 2 | 3 | 4 | 5 |
| 8 | Washing your whole body? | 1 | 2 | 3 | 4 | 5 |
| 9 | Getting dressed? | 1 | 2 | 3 | 4 | 5 |
| 10 | Dealing with people you do not know? | 1 | 2 | 3 | 4 | 5 |
| 11 | Maintaining a friendship? | 1 | 2 | 3 | 4 | 5 |
| 12 | Your day-to-day work/school? | 1 | 2 | 3 | 4 | 5 |
|  | | | | | | |
| 13 | Overall, in the past 30 days, how many days were these difficulties present? | | | ***Record number of days*** ____ | | |
| 14 | In the past 30 days, for how many days were you totally unable to carry out your usual activities or work because of any health condition? | | | ***Record number of days*** ____ | | |
| 15 | In the past 30 days, not counting the days that you were totally unable, for how many days did you cut back or reduce your usual activities or work because of any health condition? | | | ***Record number of days*** ____ | | |

| **Part FOUR: Assessment of Quality of Life of NTD Patients** |
| --- |

This assessment asks how you feel about your quality of life, health, or other areas of your life. Please answer all the questions. If you are unsure about which response to give to a question, please choose the one that appears most appropriate. This can often be your first response.

Please keep in mind your standards, hopes, pleasures and concerns. Please before answering each question, we ask that you think about your life in the **last two weeks**.*(Please circle number of the selected option for each question)*

|  |  | Very poor | Poor | Neither poor nor good | Good | Very good |
| --- | --- | --- | --- | --- | --- | --- |
| 1 | How would you rate your quality of life? | 1 | 2 | 3 | 4 | 5 |

|  |  | Very dissatisfied | Dissatisfied | Neither satisfied nor dissatisfied | Satisfied | Very Satisfied |
| --- | --- | --- | --- | --- | --- | --- |
| 2 | How satisfied are you with your health? | 1 | 2 | 3 | 4 | 5 |

|  |  | Not at all | A little | A moderate amount | Very much | An extreme amount |
| --- | --- | --- | --- | --- | --- | --- |
| 3 | To what extent do you feel that (physical)pain prevents you from doing what you need to do? | 1 | 2 | 3 | 4 | 5 |
| 4 | How much do you need any medical treatment to function in your daily life? | 1 | 2 | 3 | 4 | 5 |
| 5 | How much do you enjoy life? | 1 | 2 | 3 | 4 | 5 |
| 6 | To what extent do you feel your life to be meaningful? | 1 | 2 | 3 | 4 | 5 |

|  |  | Not at all | A little | A moderate amount | Very much | An extreme amount |
| --- | --- | --- | --- | --- | --- | --- |
| 7 | How well are you able to concentrate? | 1 | 2 | 3 | 4 | 5 |
| 8 | How safe do you feel in your daily life? | 1 | 2 | 3 | 4 | 5 |
| 9 | How healthy is your physical environment? | 1 | 2 | 3 | 4 | 5 |

|  |  | Not at all | A little | A moderate amount | Very much | An extreme amount |
| --- | --- | --- | --- | --- | --- | --- |
| 10 | Do you have enough energy for everyday life? | 1 | 2 | 3 | 4 | 5 |
| 11 | Are you able to accept your bodily appearance? | 1 | 2 | 3 | 4 | 5 |
| 12 | Have you enough money to meet your to meet your needs | 1 | 2 | 3 | 4 | 5 |
| 13 | How available to you is the information that you need in your day-to-day life? | 1 | 2 | 3 | 4 | 5 |
| 14 | To what extent do you have the opportunity for leisure activities? | 1 | 2 | 3 | 4 | 5 |

|  |  | Very poor | Poor | Neither poor nor good | Good | Very good |
| --- | --- | --- | --- | --- | --- | --- |
| 15 | How well are you able to get around? | 1 | 2 | 3 | 4 | 5 |

|  |  | Very dissatisfied | Dissatisfied | Neither satisfied nor dissatisfied | Satisfied | Very Satisfied |
| --- | --- | --- | --- | --- | --- | --- |
| 16 | How satisfied are you with your sleep? | 1 | 2 | 3 | 4 | 5 |
| 17 | How satisfied are you with your ability to perform your daily living activities? | 1 | 2 | 3 | 4 | 5 |
| 18 | How satisfied are you with your capacity for work? | 1 | 2 | 3 | 4 | 5 |
| 19 | How satisfied are you with yourself? | 1 | 2 | 3 | 4 | 5 |
| 20 | How satisfied are you with your personal relationships? | 1 | 2 | 3 | 4 | 5 |
| 21 | How satisfied are you with your sex life? | 1 | 2 | 3 | 4 | 5 |
| 22 | How satisfied are you with the support you get from your friends? | 1 | 2 | 3 | 4 | 5 |
| 23 | How satisfied are you with the conditions of your living place? | 1 | 2 | 3 | 4 | 5 |
| 24 | How satisfied are you with your access to health services? | 1 | 2 | 3 | 4 | 5 |
| 25 | How satisfied are you with your transport? | 1 | 2 | 3 | 4 | 5 |

|  |  | Never | Seldom | Quite often | Very often | Always |
| --- | --- | --- | --- | --- | --- | --- |
| 26 | How often do you have negative feelings such as blue mood, despair, anxiety, depression? | 1 | 2 | 3 | 4 | 5 |

Do you have any comments about the interview? ............................................................................................................................................................

THANK YOU FOR YOUR HELP
