# Supplementary material for: Effectiveness of self-care interventions for integrated morbidity management of skin neglected tropical diseases in Anambra State, Nigeria
Source: BMC Public Health. 2021 Sep 25;21:1748. doi: 10.1186/s12889-021-11729-1 (PMC8465703; doi:10.1186/s12889-021-11729-1)
Supplement: Supplementary file 3 — Additional file 3: Table S1. Access to water supply and environmental sanitation at baseline. [file 12889_2021_11729_MOESM3_ESM.docx]

**Additional File 3: Table S1**

**Table S1. Participant’s access to water supply and environmental sanitation at baseline**

| Variables | **n (N = 48)** | % |
| --- | --- | --- |
| Source of water for cooking |  |  |
| River | 5 | 10.4 |
| Well | 1 | 2.1 |
| Borehole | 42 | 87.5 |
|  |  |  |
| Source of water for drinking |  |  |
| River | 2 | 4.2 |
| Borehole | 44 | 91.7 |
| Other | 2 | 4.2 |
|  |  |  |
| Source of water for cleaning and washing |  |  |
| River | 7 | 14.6 |
| Well | 4 | 8.3 |
| Borehole | 37 | 77.1 |
|  |  |  |
| Able to fetch water for domestic use |  |  |
| Yes | 24 | 50 |
| No/I don’t know | 24 | 50 |
|  |  |  |
| Have someone who assists in fetching water |  |  |
| Yes | 31 | 64.6 |
| No /Don’t know | 17 | 35.4 |
|  |  |  |
| Have someone to fetch water whenever it is needed |  |  |
| Yes | 40 | 83.3 |
| No /Don’t know | 8 | 16.7 |
|  |  |  |
| Means of refuse disposal in the community |  |  |
| Dumped in community refuse collection site | 36 | 75.0 |
| Dumped in a pit in the house | 2 | 4.2 |
| Dumped at the back of the house | 9 | 18.7 |
| Others | 1 | 2.1 |
|  |  |  |
| Toilet facility |  |  |
| Water system at home | 34 | 70.8 |
| Pit-latrine at home | 9 | 18.8 |
| Community pit-latrine | 1 | 2.1 |
| Free range in the bush | 4 | 8.3 |
