# Supplementary material for: Effectiveness of self-care interventions for integrated morbidity management of skin neglected tropical diseases in Anambra State, Nigeria
Source: BMC Public Health. 2021 Sep 25;21:1748. doi: 10.1186/s12889-021-11729-1 (PMC8465703; doi:10.1186/s12889-021-11729-1)
Supplement: Supplementary file 4 — Additional file 4: Table S2. Baseline economic cost and burden to participants/family with NTDs and related impairments. [file 12889_2021_11729_MOESM4_ESM.docx]

**Additional File 4: Table S2**

**Table S2. Baseline economic cost and burden to participants/family with NTDs and related impairments**

| **Variable** | **n (N = 48)** | **%** |
| --- | --- | --- |
| **Self-reported limitation due to disease** |  |  |
| Yes | 35 | 72.9 |
| No | 13 | 27.1 |
| **Severity of limitation due to disease** |  |  |
| Not limited | 13 | 27.1 |
| Mildly limited | 0 | 0 |
| Moderately limited | 14 | 29.2 |
| Severely limited | 21 | 43.8 |
| **Changed job due to disease** |  |  |
| Yes | 11 | 22.9 |
| No | 37 | 77.1 |
| **Mean earnings loss per month (US$)** | 10.80 |  |
| **Loss of work time in the last month due to disease** |  |  |
| Yes | 23 | 47.9 |
| No | 25 | 52.1 |
| **Mean lost work days per month (days)** | 9.50 |  |
| **Reason for any loss of work time in the last month** |  |  |
| None | 14 | 29.2 |
| Treatment-seeking | 19 | 39.6 |
| Pain | 14 | 29.2 |
| Reaction what does this mean? | 1 | 2.1 |
| **Hours of work per day** |  |  |
| 0 | 26 | 54.2 |
| 1 – 4 | 4 | 8.3 |
| 5 – 8 | 7 | 14.6 |
| > 8 | 11 | 22.9 |
| **Hours of work per day by** **healthy persons in the same job** |  |  |
| 0 A healthy person can do the job in zero hours? This doesn’t make sense to me. | 9 | 18.8 |
| 1 – 4 | 1 | 2.1 |
| 5 – 8 | 11 | 22.9 |
| >8 | 27 | 56.3 |
| **Time off from work by caregiver to care for participant** |  |  |
| None | 23 | 47.9 |
| Yes | 25 | 52.1 |
| **Meantime-off taken (hours)** | 4.02 |  |
|  |  |  |
| **School absenteeism among children in family of participant (days)** |  |  |
| 0 | 41 | 85.4 |
| 1 - 14 | 7 | 14.6 |
| **Caregiver days of work days lost to care for participants** |  |  |
| None (0) | 33 | 68.8 |
| 1 – 3 | 3 | 6.3 |
| > 3 | 12 | 25.0 |
| **Mean duration off work (days)** | 2.1 |  |
