# Supplementary material for: Effectiveness of self-care interventions for integrated morbidity management of skin neglected tropical diseases in Anambra State, Nigeria
Source: BMC Public Health. 2021 Sep 25;21:1748. doi: 10.1186/s12889-021-11729-1 (PMC8465703; doi:10.1186/s12889-021-11729-1)
Supplement: Supplementary file 5 — Additional file 5: Table S3. Baseline disability quality-of-life (transformed to WHOQOL-BREF 100 Scale) status of the study participants according to gender (N = 48). [file 12889_2021_11729_MOESM5_ESM.docx]

**Additional File 5: Table S3**

**Table S3. Baseline disability quality-of-life (transformed to WHOQOL-BREF 100 Scale) status of the study participants according to gender (N = 48)**

|  | **All** | **Male** | **Female** | **p-value** |
| --- | --- | --- | --- | --- |
|  | Mean (SD) | Mean (SD) | Mean (SD) |  |
| **Disability item** |  |  |  |  |
| Total score (0 – 48) | 19.9 (11.1) | 19.0 (10.6) | 20.5 (11.6) | 0.66 |
| Standing (0 – 4) | 2.0 (1.6) | 1.9 (1.6) | 2.1 (1.6) | 0.80 |
| Household tasks | 1.5 (1.5) | 1.5 (1.4) | 1.6 (1.5) | 0.88 |
| Learning | 1.8 (1.4) | 1.7 (1.3) | 1.8 (1.5) | 0.69 |
| Community life | 1.9 (1.6) | 1.7 (1.4) | 2.1 (1.6) | 0.36 |
| Emotional functions | 2.4 (1.1) | 2.1 (1.2) | 2.6 (1.1) | 0.14 |
| Concentrating | 1.7 (1.2) | 1.6 (1.4) | 1.7 (1.2) | 0.70 |
| Walking | 2.4 (1.4) | 2.2 (1.4) | 2.5 (1.5) | 0.52 |
| Washing oneself | 0.9 (1.2) | 1.0 (1.1) | 0.9 (1.3) | 0.79 |
| Dressing oneself | 0.8 (1.2) | 0.9 (1.3) | 0.8 (1.2) | 0.74 |
| Dealing with strangers | 1.3 (1.2) | 1.4 (1.5) | 1.2 (1.0) | 0.60 |
| Maintaining friendships | 1.2 (1.2) | 1.2 (1.3) | 1.2 (1.1) | 0.85 |
| Day-to-day work ability | 2.0 (1.4) | 1.9 (1.3) | 2.0 (1.4) | 0.83 |
|  |  |  |  |  |
| **Quality of Life Domain** |  |  |  |  |
| Total score (100) | 49.0 (15.0) | 48.9 (13.9) | 49.1 (15.9) | 0.96 |
| Physical domain | 45.6 (20.9) | 44.2 (21.7) | 46.4 (20.8) | 0.732 |
| Psychological domain | 45.9 (19.1) | 43.1 (19.2) | 47.6 (19.1) | 0.436 |
| Social domain | 54.2 (21.6) | 54.9 (20.5) | 53.7 (22.6) | 0.860 |
| Environmental domain | 50.5 (13.4) | 53.3 (12.6) | 48.8 (13.8) | 0.266 |
|  |  |  |  |  |
| **Disability grade** | n (%) | n (%) | n (%) |  |
| None | 1 (2.1) | 0 (0) | 1 (3.3) | 0.871 |
| Mild | 3 (6.2) | 1 (5.6) | 2 (6.7) |  |
| Moderate | 7 (14.6) | 3 (16.7) | 4 (13.3) |  |
| Severe | 37 (77.1) | 14 (77.8) | 23 (76.7) |  |
|  |  |  |  |  |
