# Supplementary material for: Effectiveness of self-care interventions for integrated morbidity management of skin neglected tropical diseases in Anambra State, Nigeria
Source: BMC Public Health. 2021 Sep 25;21:1748. doi: 10.1186/s12889-021-11729-1 (PMC8465703; doi:10.1186/s12889-021-11729-1)
Supplement: Supplementary file 6 — Additional file 6: Table S4. Differences in baseline economic burden, disability status and quality of life of participants who maintained and did not maintain self-care. [file 12889_2021_11729_MOESM6_ESM.docx]

**Additional File 6: Table S4**

**Table S4. Differences in baseline economic burden, disability status and quality of life of participants who maintained and did not maintain self care**

| **Variable** | **Self care maintained**  **N = 30** | **Stopped self care**  **N = 18** | **p-value** |
| --- | --- | --- | --- |
| Mean (SD) income (US$) | 29.65 (90.52) | 52.06 (87.04) | 0.404 |
| Mean (SD) household cost (US$) | 157.50 (180.33) | 90.79 (71.94) | 0.142 |
| Mean (SD) disability score | 22.3 (9.7) | 15.9 (12.3) | 0.051 |
| Mean (SD) quality of life score | 45.7 (15.7) | 54.5 (12.3) | 0.049 |
