# Supplementary material for: Effectiveness of self-care interventions for integrated morbidity management of skin neglected tropical diseases in Anambra State, Nigeria
Source: BMC Public Health. 2021 Sep 25;21:1748. doi: 10.1186/s12889-021-11729-1 (PMC8465703; doi:10.1186/s12889-021-11729-1)
Supplement: Supplementary file 7 — Additional file 7: Table S5. Morbidity burden of the participants before and after self-care programme, Anambra State (N = 30). [file 12889_2021_11729_MOESM7_ESM.docx]

**Additional File 7: Table S5**

**Table S5. Morbidity burden of the participants before and after self-care programme, Anambra State (N = 30)**

| **Variable** | **Baseline**  **n (%)** | **Self-care**  **n (%)** | **P- value** |
| --- | --- | --- | --- |
| **Self-reported limitation due to disease** |  |  | 0.180** |
| Yes | 26 (86.7) | 21 (70) |  |
| No | 4 (13.3) | 9 (30) |  |
| **Severity of limitation due to disease** |  |  |  |
| Not limited | 5 (16.7) | 11 (36.7) |  |
| Mildly limited | 0 (0) | 3 (10) |  |
| Moderately limited | 8 (26.7) | 4 (13.3) |  |
| Severely limited | 17 (56.7) | 12 (40) |  |
|  |  |  |  |
| **Participants who changed job due to disease** | 5 (16.7) | 7 (23.3) | 0.625** |
|  |  |  |  |
| **Mean (SD) monthly loss due to disease (US$)** | 7.53 (41.23) | 5.38 (28.25) | 0.818* |
|  |  |  |  |
| **Participants who lost work time in the last month due to disease** | 15 (50.0) | 12 (40.0) |  |
|  |  |  |  |
| **Mean (SD) monthly lost workdays due to disease** | 9.83 (12.6) | 6.9 (12.0) | 0.342* |
|  |  |  |  |
| **Reason for any loss of work time in the last month** |  |  |  |
| None | 8 (26.7) | 19 (63.3) |  |
| Treatment-seeking | 13 (43.3) | 7 (23.3) |  |
| Pain | 8 (26.7) | 4 (13.30) |  |
| Reaction (adverse effect) | 1 (3.3) | 0 (0) |  |
| **Hours of work per day in the previous month** |  |  | 0.416** |
| 0 | 18 (60.0) | 14 (46.7) |  |
| 1 – 4 | 3 (10.0) | 1 (3.3) |  |
| 5 – 8 | 5 (16.7) | 9 (30) |  |
| > 8 | 4 (13.3) | 6 (20) |  |
|  |  |  | – |
| **Time taken by caregiver to care for participant in the last month** |  |  |  |
| None | 9 (30.0) | 17 (56.7) |  |
| Yes | 21 (70.0) | 13 (43.3) |  |
| **Mean (SD) time taken (hours) for caregiving.** | 6.1 (7.3) | 0.5 (1.2) | <0.001* |
|  |  |  |  |
| **Caregiver days off work to care for participants** |  |  |  |
| Mean (SD) duration off work (days) | 3.07 (5.1) | 0.1 (0.7) | 0.005* |

**P –value based on McNemar’s Chi-Square test

*p- value based on paired t-test.
