# Supplementary material for: Effectiveness of self-care interventions for integrated morbidity management of skin neglected tropical diseases in Anambra State, Nigeria
Source: BMC Public Health. 2021 Sep 25;21:1748. doi: 10.1186/s12889-021-11729-1 (PMC8465703; doi:10.1186/s12889-021-11729-1)
Supplement: Supplementary file 8 — Additional file 8: Table S6. Costs incurred by participants at baseline and after self-care, Anambra State (N = 30). [file 12889_2021_11729_MOESM8_ESM.docx]

Additional File 8: Table S6

Table S6. Costs incurred by participants at baseline and after self-care, Anambra State (N = 30)

|  | Baseline (US$) | |  | After Self-care (US$) | | p -value | |
| --- | --- | --- | --- | --- | --- | --- | --- |
|  | n (%) | Mean (SD) |  | n (%) | Mean (SD) | (based on paired *t*-test) | |
| OUT-OF-POCKET COSTS |  |  |  |  |  |  |  |
| Clean water for chores and self-care | 11 (36.7) | 3.55 (5.53) |  | 3 (10) | 0.22 (0.70) | 0.003 |  |
| Getting persons to assist with your work | 7 (23.3) | 4.68 (10.85) |  | 2 (6.7) | 1.29 (6.22) | 0.043 |  |
| Getting persons to care for your children | 0 (0) | 0 (0) |  | 0 (0) | 0 (0) | – |  |
| Transportation costs for health-seeking | 23 (76.7) | 9.44 (13.12) |  | 10 (33.3) | 1.76 (5.75) | 0.003 |  |
| Transportation costs to work/school | 3 (10.0) | 0.20 (0.76) |  | 2 (6.7) | 1.02 (5.30) | 0.413 |  |
| Transportation costs to/from market | 6 (20.0) | 1.37 (4.01) |  | 5 (16.7) | 0.32 (0.77) | 0.166 |  |
| Transportation costs to/from events | 10 (33.3) | 1.93 (3.43) |  | 7 (23.3) | 0.67 (1.45) | 0.067 |  |
| Self-care materials | 22 (73.3) | 2.65 (2.43) |  | 18 (60) | 1.45 (1.76) | 0.017 |  |
| Wound care materials | 19 (63.3) | 6.77 (7.98) |  | 15 (50) | 5.44 (9.32) | 0.399 |  |
| Care from traditional healers | 10 (33.3) | 60.51 (154.14) |  | 2 (6.7) | 4.84 (21.24) | 0.063 |  |
| Paying a caregiver/help | 7 (23.3) | 3.85 (7.93) |  | 2 (6.7) | 0.32 (1.23) | 0.017 |  |
| Paying for painkillers | 23 (76.7) | 10.24 (19.28) |  | 17 (56.7) | 4.20 (6.42) | 0.100 |  |
|  |  |  |  |  |  |  |  |
| To pay for antibiotics | 11 (36.7) | 3.35 (6.91) |  | 8 (26.7) | 4.43 (14.96) | 0.599 |  |
| To pay for antifungal | 1 (3.3) | 0.04 (0.21) |  | 1 (3.3) | 0.16 (0.88) | 0.546 |  |
| To obtain special footwear / clothing | 2 (6.7) | 0.34 (1.35) |  | 3 (10) | 0.61 (1.96) | 0.465 |  |
|  |  |  |  |  |  |  |  |
| Mobility assistance / devices | 3 (10.0) | 1.40 (4.69) |  | 2 (6.7) | 0.58 (2.09) | 0.318 |  |
|  |  |  |  |  |  |  |  |
| Average Out-of-pocket cost | 30 (100) | 110.30 (163.44) |  | 30 (100) | 27.28 (32.60) | 0.010 |  |
|  |  |  |  |  |  |  |  |
| EARNINGS LOSS |  |  |  |  |  |  |  |
| Mean (SD) income loss due to job change | 30 (100) | 7.53 (41.23) |  | 30 (100) | 5.38 (28.25) | 0.818 |  |
| Mean (SD) income lost due to inability to work in the previous month | 30 (100) | 28.55 (36.60) |  | 30 (100) | 20.03 (32.50) | 0.342 |  |
| Earnings loss |  | 36.08 (9.30) |  |  | 25.41 (7.51) | 0.395 |  |
|  |  |  |  |  |  |  |  |
| PARTICIPANT COSTS |  | 146.38 (178.58) |  |  | 52.69 (53.94) | 0.008 |  |
|  |  |  |  |  |  |  |  |
| CAREGIVER COSTS |  |  |  |  |  |  |  |
| Mean (SD) value of time-off taken, and forgone income by carer due to days off work to care for participant |  | 11.12 (16.28) |  |  | 0.56 (2.13) | 0.002 |  |
|  |  |  |  |  |  |  |  |
| HOUSEHOLD COSTS |  | 157.50 (180.33) |  |  | 53.24 (54.57) | 0.004 |  |

1US$ = 310 Naira;
