# Supplementary material for: Effectiveness of self-care interventions for integrated morbidity management of skin neglected tropical diseases in Anambra State, Nigeria
Source: BMC Public Health. 2021 Sep 25;21:1748. doi: 10.1186/s12889-021-11729-1 (PMC8465703; doi:10.1186/s12889-021-11729-1)
Supplement: Supplementary file 9 — Additional file 9: Table S7. Association between household costs and demographic characteristics of the participants (N = 30). [file 12889_2021_11729_MOESM9_ESM.docx]

**Additional File 9: Table S7**

**Table S7. Association between household costs and demographic characteristics of the participants (N = 30)**

| **Variables** | **Baseline costs (US$)** | | |  | | **Costs after self-care (US$)** | | |
| --- | --- | --- | --- | --- | --- | --- | --- | --- |
|  | Mean (SD) | p - value |  | | Mean (SD) | | p - value |  |
| Age group (years) |  | 0.631 |  | |  | | 0.741 |  |
| ≤ 20 | 60.30 (28.9) |  |  | | 30.9 (54.7) | |  |  |
| 21 – 40 | 180.80(212.0) |  |  | | 50.5 (61.1) | |  |  |
| 41 – 60 | 190.20 (209.0) |  |  | | 57.2 (41.8) | |  |  |
| ≥ 61 | 122.5 (95.0) |  |  | | 72.0 (63.8) | |  |  |
|  |  |  |  | |  | |  |  |
| Gender |  | 0.393 |  | |  | | 0.117 |  |
| Male | 192.7 (139.2) |  |  | | 72.4 (69.7) | |  |  |
| Female | 134.1 (97.2) |  |  | | 40.4 (38.7) | |  |  |
|  |  |  |  | |  | |  |  |
| Religion |  | 0.011 |  | |  | | 0.772 |  |
| Catholic | 126.6 (187.0) |  |  | | 49.3 (63.0) | |  |  |
| Protestant | 121.1 (81.3) |  |  | | 49.8 (46.1) | |  |  |
| Traditional religion | 681.0 (0) |  |  | | 106.5 (0) | |  |  |
| Other | 235.0 (179.1) |  |  | | 63.1 (57.6) | |  |  |
|  |  |  |  | |  | |  |  |
| Marital status |  | 0.592 |  | |  | | 0.800 |  |
| Married | 197.5 (211.4) |  |  | | 54.1 (61.2) | |  |  |
| Never married | 99.5 (135.6) |  |  | | 54.1 (48.7) | |  |  |
| Separated | 80.3 (0) |  |  | | 104.0 (0) | |  |  |
| Widowed | 131.0 (88.2) |  |  | | 55.9 (45.7) | |  |  |
|  |  |  |  | |  | |  |  |
| Education |  | 0.955 |  | |  | | 0.200 |  |
| No formal education | 117.5 (36.3) |  |  | | 109.3 (32.3) | |  |  |
| Primary | 172.4 (236.7) |  |  | | 53.3 (54.7) | |  |  |
| Secondary | 152.9 (186.7) |  |  | | 48.9 (55.4) | |  |  |
| Tertiary | 206.5 (11.4) |  |  | | 7.9 (11.2) | |  |  |
|  |  |  |  | |  | |  |  |
| Occupation |  | 0.169 |  | |  | | 0.268 |  |
| Employed | 227.6 (243.2) |  |  | | 58,6 (62.8) | |  |  |
| Unemployed | 127.5 (115.8) |  |  | | 60.5 (51.3) | |  |  |
| Student | 51.9 (24.6) |  |  | | 11.2 (16.3) | |  |  |
|  |  |  |  | |  | |  |  |
| Household income |  | 0.001 |  | |  | | 0.661 |  |
| No defined income | 120.2 (109.3) |  |  | | 57.5 (58.6) | |  |  |
| Irregular income | 169.0 (196.5) |  |  | | 49.8 (49.3) | |  |  |
| Regular income | 751.6 (0) |  |  | | 6.9 (0) | |  |  |
|  |  |  |  | |  | |  |  |
| Participant’s diagnosis |  | 0.381 |  | |  | | 0.251 |  |
| Buruli ulcer | 165.4 (184.0) |  |  | | 56.3 (55.2) | |  |  |
| Lymphatic filariasis | 47.4 (56.4) |  |  | | 9.8 (10.3) | |  |  |

1US$ = N310.
