# Supplementary material for: Effectiveness of self-care interventions for integrated morbidity management of skin neglected tropical diseases in Anambra State, Nigeria
Source: BMC Public Health. 2021 Sep 25;21:1748. doi: 10.1186/s12889-021-11729-1 (PMC8465703; doi:10.1186/s12889-021-11729-1)
Supplement: Supplementary file 10 — Additional file 10: Table S8. Association between total disability status and demographic profile of the participants (N = 30). [file 12889_2021_11729_MOESM10_ESM.docx]

**Additional File 10: Table S8**

**Table S8. Association between total disability status and demographic profile of the participants (N = 30)**

| **Variables** | **Baseline disability status** | |  | **Disability status after self care** | | |
| --- | --- | --- | --- | --- | --- | --- |
|  | Mean (SD) | p - value |  | Mean (SD) | p – value |  |
| Age group (years) |  | 0.331 |  |  | 0.004 |  |
| ≤ 20 | 15.3 (11.5) |  |  | 3.0 (2.9) |  |  |
| 21 – 40 | 21.5 (10.6) |  |  | 10.8 (7.3) |  |  |
| 41 – 60 | 25.1 (9.0) |  |  | 20.8 (8.6) |  |  |
| ≥ 61 | 25.8 (9.7) |  |  | 11.2 (8,2) |  |  |
|  |  |  |  |  |  |  |
| Gender |  | 0.172 |  |  | 0.917 |  |
| Male | 19.3 (8.3) |  |  | 12.3 (8.3) |  |  |
| Female | 24.3 (10.3) |  |  | 12.6 (9.8) |  |  |
|  |  |  |  |  |  |  |
| Religion |  | 0.344 |  |  | 0.711 |  |
| Catholic | 18.9 (10.7) |  |  | 10.8 (9.0) |  |  |
| Protestant | 25.8 (8.6) |  |  | 15.1 (10.3) |  |  |
| Traditional religion | 25.0 (0) |  |  | 12.0 (0) |  |  |
| Other | 24.3 (8.1) |  |  | 11.3 (7.8) |  |  |
|  |  |  |  |  |  |  |
| Marital status |  | 0.816 |  |  | 0.036 |  |
| Married | 23.5 (10.2) |  |  | 12.4 (7.8) |  |  |
| Never married | 20.7 (10.5) |  |  | 8.3 (8.0) |  |  |
| Separated | 27.0 (0) |  |  | 32.0 (0) |  |  |
| Widowed | 19.3 (7.5) |  |  | 19.0 (11.5) |  |  |
|  |  |  |  |  |  |  |
| Education |  |  |  |  | 0.230 |  |
| No formal education | 27.3 (0.58) |  |  | 22.7 (9.5) |  |  |
| Primary | 24.7 (4.4) |  |  | 11.1 (9.5) |  |  |
| Secondary | 20.1 (11.3) |  |  | 11.7 (8.4) |  |  |
| Tertiary | 27.0 (14.1) |  |  | 9.0 (9.1) |  |  |
|  |  |  |  |  |  |  |
| Occupation |  | 0.052 |  |  | 0.067 |  |
| Employed | 19.8 (8.7) |  |  | 13.9 (8.7) |  |  |
| Unemployed (health reasons) | 26.6 (8.9) |  |  | 14.0 (9.2) |  |  |
| Student | 15.0 (11.2) |  |  | 2.8 (2.8) |  |  |
|  |  |  |  |  |  |  |
| Household income |  | 0.479 |  |  | 0.963 |  |
| No defined income | 21.6 (10.2) |  |  | 12.5 (9.1) |  |  |
| Irregular income | 22.5 (9.1) |  |  | 12.7 (10.1) |  |  |
| Regular income | 34.0 (0) |  |  | 10.0 (0) |  |  |
|  |  |  |  |  |  |  |
| Participant’s diagnosis |  | 0.540 |  |  | 0.383 |  |
| Buruli ulcer | 22.0 (9.5) |  |  | 12.1 (9.1) |  |  |
| Lymphatic filariasis | 26.5 (16.3) |  |  | 18.0 (9.9) |  |  |
