# Supplementary material for: Effectiveness of self-care interventions for integrated morbidity management of skin neglected tropical diseases in Anambra State, Nigeria
Source: BMC Public Health. 2021 Sep 25;21:1748. doi: 10.1186/s12889-021-11729-1 (PMC8465703; doi:10.1186/s12889-021-11729-1)
Supplement: Supplementary file 11 — Additional file 11: Table S9. Association between total HRQoL and demographic profile of the participants (N = 30). [file 12889_2021_11729_MOESM11_ESM.docx]

**Additional File 11: Table S9**

**Table S9. Association between total HRQoL and demographic profile of the participants (N = 30)**

|  | **Baseline HRQoL** | |  | **HRQoL after self care** | |
| --- | --- | --- | --- | --- | --- |
| **Variables** | Mean (SD) | p - value |  | Mean (SD) | p - value |
| Age group (years) |  | 0.926 |  |  | 0.020 |
| ≤ 20 | 49.3 (19.9) |  |  | 72.4 (9.5) |  |
| 21 – 40 | 46.4 (19.7) |  |  | 56.6 (56.3) |  |
| 41 – 60 | 45.3 (9.3) |  |  | 47.4 (50.9) |  |
| ≥ 61 | 42.0 (12.9) |  |  | 64.7 (7.2) |  |
|  |  |  |  |  |  |
| Gender |  | 0.555 |  |  | 0.491 |
| Male | 47.9 (11.7) |  |  | 59.9 (15.2) |  |
| Female | 44.3 (18.2) |  |  | 56.0 (15.0) |  |
|  |  |  |  |  |  |
| Religion |  | 0.464 |  |  | 0.225 |
| Catholic | 49.1 (16.9) |  |  | 62.3 (12.1) |  |
| Protestant | 44.1 (15.3) |  |  | 50.1 (15.3) |  |
| Traditional religion | 56.3 (0) |  |  | 64.3 (0) |  |
| Other | 36.2 (12.6) |  |  | 59.5 (20.2) |  |
|  |  |  |  |  |  |
| Marital status |  | 0.708 |  |  | 0.015 |
| Married | 48.5 (15.7) |  |  | 59.3 (12.3) |  |
| Never married | 43.7 (18.9) |  |  | 61.4 (14.4) |  |
| Separated | 37.5 (0) |  |  | 35.8 (0) |  |
| Widowed | 39.1 (4.6) |  |  | 50.1 (13.3) |  |
|  |  |  |  |  |  |
| Education |  | 0.30 |  |  | 0.358 |
| No formal education | 42.3 (10.9) |  |  | 43.9 (24.4) |  |
| Primary | 39.0 (11.9) |  |  | 61.1 (12.2) |  |
| Secondary | 50.1 (15.1) |  |  | 57.6 (14.5) |  |
| Tertiary | 35.1 (36.6) |  |  | 64.4 (6.5) |  |
|  |  |  |  |  |  |
| Occupation |  | 0.144 |  |  | 0.153 |
| Employed | 51.0 (12.0) |  |  | 54.5 (18.5) |  |
| Unemployed | 39.7 (16.8) |  |  | 56.3 (10.4) |  |
| Student | 51.2 (18.1) |  |  | 70.8 (11.9) |  |
|  |  |  |  |  |  |
| Household income |  | 0.30 |  |  | 0.886 |
| No defined income | 46.0 (15.6) |  |  | 56.5 (13.4) |  |
| Irregular income | 43.5 (16.6) |  |  | 59.3 (18.7) |  |
| Regular income | 62.5 (0) |  |  | 59.5 (0) |  |
|  |  |  |  |  |  |
| Participant’s diagnosis |  | 0.909 |  |  | 0.630 |
| Buruli ulcer | 45.7 (16.3) |  |  | 57.9 (15.0) |  |
| Lymphatic filariasis | 47.0 (6.7) |  |  | 52.5 (18.7) |  |
