# Supplementary material for: Effectiveness of self-care interventions for integrated morbidity management of skin neglected tropical diseases in Anambra State, Nigeria
Source: BMC Public Health. 2021 Sep 25;21:1748. doi: 10.1186/s12889-021-11729-1 (PMC8465703; doi:10.1186/s12889-021-11729-1)
Supplement: Supplementary file 12 — Additional file 12: Table S10. Changes in economic burden, disability status and quality of life of NTD and non-NTD patients who completed self-care. [file 12889_2021_11729_MOESM12_ESM.docx]

**Additional File 12: Table S10**

**Table S10. Changes in economic burden, disability status and quality of life of NTD and non-NTD patients who completed self-care**

| **Variable** | **NTDs (N = 21)** | **Non-NTDs (N=9)** | ***p-value** |
| --- | --- | --- | --- |
| Mean (SD) Income (US$) | 38.5 (± 106.4) | 9.9 (± 26.9) | 0.422 |
|  |  |  |  |
| Mean (SD) household cost (US$) |  |  |  |
| Baseline | 173.4 (± 208.9) | 120.3 (± 80.7) | 0.469 |
| Endline | 48.8 (± 47.7) | 63.7 (± 70.2) | 0.501 |
|  |  |  |  |
| Mean (SD) disability score |  |  |  |
| Baseline | 21.1 (± 9.6) | 25.3 (± 9.9) | 0.277 |
| Endline | 10.1 (± 8.6) | 18.0 (± 8.0) | 0.026 |
|  |  |  |  |
| Mean (SD) quality of life score |  |  |  |
| Baseline | 47.6 (± 17.4) | 41.4 (±10.7) | 0.328 |
| Endline | 62.8 (± 13.7) | 45.2 (± 9.8) | 0.002 |

*p-value based on t-test; SD = standard deviation
